# Insights into the roles of lncRNAs in skeletal and dental diseases

**DOI:** 10.1186/s13578-018-0208-4

**Published:** 2018-02-05

**Authors:** Yuyu Li, Jiawei Zhang, Jie Pan, Xu Feng, Peipei Duan, Xing Yin, Yang Xu, Xin Wang, Shujuan Zou

**Affiliations:** 1 State Key Laboratory of Oral Diseases, National Clinical Research Center for Oral Diseases, West China Hospital of Stomatology, Sichuan University, No.14, 3rd Section, Renmin South Road, Chengdu, 610041 China; 2 Department of Orthodontics, West China Hospital of Stomatology, Sichuan University, No.14, 3rd Section, Renmin South Road, Chengdu, 610041 China; 3 Department of Pathology, University of Alabama at Birmingham, 1670 University Blvd., VH G019E, Birmingham, AL 35294 USA

**Keywords:** lncRNA, Osteoblastogenesis, Osteoclastogenesis, Skeletal and dental diseases

## Abstract

Long noncoding RNAs (lncRNAs) are a class of non-protein-coding transcripts with the length longer than 200 nucleotides. Growing evidence suggests that lncRNAs, which were initially thought to be merely transcriptional “noise”, participate in a wide repertoire of biological processes. It has been well established that lncRNAs not only play important roles in genomic regulation, transcription, posttranscriptional processes but are also implicated in the pathogenesis of human diseases including cardiovascular diseases, diabetes, neurodegenerative disorders, and cancer. However, the pathological role of lncRNAs in skeletal and dental diseases is just beginning to be uncovered. In the present review, we outline the current understanding of the established functions and underlying mechanisms of lncRNAs in various cellular processes. Furthermore, we discuss new findings on the role of lncRNAs in osteoblastogenesis and osteoclastogenesis as well as their involvement in skeletal and dental diseases. This review intends to provide a general framework for the actions of lncRNAs and highlight the emerging evidence for the functions of lncRNAs in skeletal and dental diseases.

## Background

The term noncoding RNA (ncRNA) refers to diverse RNA molecules that do not encode any protein. ncRNAs include infrastructural RNAs such as transfer RNAs (tRNAs), ribosomal RNAs (rRNAs), small nuclear RNAs (snRNAs), and small nucleolar RNAs (snoRNAs) [1]. Increasing evidence indicates that additional regulatory ncRNAs such as lncRNAs exist and play important roles in regulating chromatin architecture/epigenetic memory, transcription, and mRNA splicing, stability, and translation [2].

lncRNAs are among the least understood RNAs despite their pervasive transcription in the genome. The most updated annotation of the human genome (Version 27, GRCh38) identifies 27,908 lncRNA transcripts from 15,778 lncRNA genes (http://www.gencodegenes.org/stats/current.html). Like mRNAs, many polyadenylated lncRNAs are transcribed by RNA polymerase II (Pol II) and are often alternatively spliced into multiple isoforms. But genes coding for lncRNAs also have characteristics distinct from those for mRNAs: lncRNA genes have fewer but longer exons, tend to be expressed at lower levels, and exhibit less-conserved sequences [3]. However, some lncRNAs that are expressed in development- and tissue-specific patterns have highly conserved promoter regions and splice sites [4].

lncRNAs do not possess any apparent protein-coding potential and are mostly expressed at low levels; they are thus characterized largely by bioinformatic approaches. Advances in high-throughput RNA sequencing technologies provide systems with which RNA transcription can be observed in an unbiased manner [5]. lncRNAs have been shown to regulate various biological processes via distinct mechanisms [6], whereas mutations or aberrant expression of lncRNAs have been implicated in the pathogenesis of a wide range of human diseases [7–9].

In this review, we briefly present the current knowledge of the functions and mechanisms of lncRNAs. We then review the role of lncRNAs in osteoblastogenesis and osteoclastogenesis based on data from multiple studies. Finally, we discuss the important roles of lncRNAs in the etiology of skeletal and dental disorders.

## Functions and mechanisms of lncRNAs

The discovery of the myriad roles of lncRNAs has made it increasingly clear that lncRNAs can function via numerous paradigms and are key regulatory molecules in cells [6]. They not only participate in nuclear events such as chromatin modification and transcription [10], but also reside in the cytoplasm, where they interact with RNA-binding proteins or modulate mRNA translation. Here, we outline a concise scheme of the functions of lncRNAs for a better understanding of what roles lncRNAs play in osteoblastogenesis, osteoclastogenesis, as well as skeletal and dental diseases.

### lncRNAs in chromatin modification

**Fig. 1:**
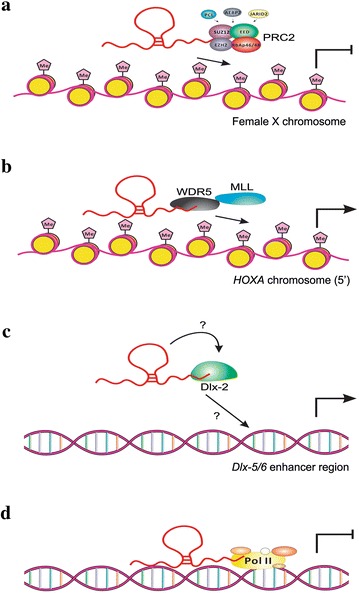
Schematic representation of how lncRNAs may function in genome regulation. **a** The participation of lncRNA *XIST* in chromosome silencing. *XIST* recruits PRC2 to the female X chromosome, leading to H3K27me3 formation (presented as pink pentagon with “Me”) and silencing of the chromosome. **b** Chromatin modification by lncRNA *HOTTIP*. *HOTTIP* interacts with adaptor protein WDR5 and targets WDR5/MLL complexes, inducing H3K4me3 (presented as a pink pentagon with “Me”) and transcription of 5′*HOXA* genes. **c** *Evf*-*2* as an example of lncRNAs that facilitate transcriptional activation. Combination of *Evf*-*2* with the protein Dlx-2 forms an *Evf*-*2*/Dlx-2 complex, which targets the *Dlx*-*5/6* enhancer region and promotes transcription. Question marks indicate that the specific role of *Evf*-*2* in the process remains to be elucidated. **d** *Alu* ncRNA can act as a potent transcriptional repressor. *Alu* RNA contains RNA polymerase II (Pol II, enzyme that synthesizes mRNAs in eukaryotes) binding arms and modular repression domains, allowing it to bind Pol II and block RNA synthesis

In the nucleus, lncRNAs target some chromatin remodeling complexes and guide them to specific genomic loci, leading to changes in gene transcription. A classic example is a long intergenic noncoding RNA (lincRNA) termed X inactive-specific transcript (*XIST*), which is transcribed from one of the two X chromosomes in female mammals (Fig. 1a). It recruits polycomb group complexes, such as polycomb repressive complex 2 (PRC2) [11], to the female X chromosome, leading to transcriptional silencing *in cis* across a majority of the chromosome. In contrast, lncRNA *HOTAIR*, which is transcribed from the antisense strand of homeobox C (*HOXC*) locus, recruits PRC2 *in trans* to the *HOXD* cluster for epigenetic repression [12].

lncRNAs also associate with other chromatin regulators. An example is the lineage-specific imprinting mediated by the lncRNA *Kcnq1ot1*, a nuclear and moderately stable transcript from the paternal chromosome. In addition to interacting with members of the PRC2 complex, *Kcnq1ot1* also recruits chromatin regulators such as G9a methyltransferase to mediate repressive histone modifications, including the trimethylation of lysine 27 on histone H3 (H3K27me3) and trimethylation of lysine 9 on histone H3 (H3K9me3) in the *Kcnq1* domain [13].

However, some lncRNAs function in chromatin activation rather than chromatin silencing. Enhancers are regulatory elements that increase the expression of target genes [14]. An enhancer-like lncRNA termed *HOTTIP* has been identified as a key intermediate that transmits information from higher order chromosomal looping into chromatin modifications. *HOTTIP* is transcribed from the distal 5′tip of the *HOXA* locus and is brought into close proximity to multiple *HOXA* genes by chromosomal looping of the *HOXA* 5′end. It directly binds the adaptor protein WDR5 and targets WDR5/MLL complexes across the *HOXA* locus, leading to histone H3 lysine 4 trimethylation (H3K4me3) and gene transcription [15] (Fig. 1b).

### lncRNAs in transcription regulation

As shown above, lncRNAs indirectly influence transcription through chromatin modification. However, some lncRNAs regulate transcription directly. The 3.8-kb lncRNA *Evf*-*2* is transcribed from the *Dlx*-*5/6* ultraconserved region. *Evf*-*2* has been found to activate the transcriptional activity of the *Dlx*-*5/6* enhancer by cooperating with a homeodomain protein Dlx-2 (Fig. 1c). This single-stranded RNA and the ultraconserved protein form an *Evf*-*2*/Dlx-2 complex and then the complex targets the *Dlx*-*5/6* enhancer. But whether the complex helps Dlx-2 bind to the enhancer site or only helps stabilize the protein requires further investigation [16]. Nevertheless, some lncRNAs serve as transcriptional repressors [6]. In response to heat shock, the *Alu* ncRNA binds Pol II and enters complexes at promoters, ultimately blocking all detectable RNA synthesis (Fig. 1d). An interesting thing is that although there are sequence discrepancies between the Pol II-binding domains of *Alu* RNA and B2 RNA (*Alu* RNA-like sequences in mouse), both of them repress transcription by Pol II. So the mechanism by which *Alu* RNA functions may not be sequence specific [17].

### lncRNAs in pre-mRNA splicing

**Fig. 2:**
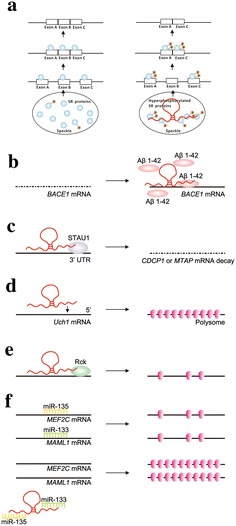
A schematic diagram illustrating the involvement of lncRNAs in posttranscriptional processes. **a** lncRNA *MALAT1* is implicated in pre-mRNA splicing. *MALAT1* changes the expression and ratio of phosphorylated (orange circle with the letter “p”) versus dephosphorylated serine/arginine-rich (SR) proteins (splicing factors can regulate splicing; blue circle), thus altering the splicing mode of pre-mRNAs. **b** lncRNAs also influence mRNA stability. lncRNA *BACE1*-*AS* associates with cell stressor Aβ 1–42 and stabilizes *BACE1* mRNA (dashed line represents unstable mRNA). **c** lncRNAs may cause mRNA decay. lncRNA *1/2*-*sbsRNA* mediates mRNA decay by binding protein STAU1, with further base-pairing with an Alu element at the 3′-untranslated regions (3′-UTRs) of *CDCP1* or *MTAP* mRNA (dashed line represents unstable mRNA). lncRNAs can serve as activators **d** or repressors **e** in mRNA translation. *Antisense Uchl1* RNA associates mRNA with active polysomes, resulting in the promotion of translation. Conversely, *lincRNA*-*p21* enhances interaction between translational repressor Rck and mRNAs such as *CTNNB1* and *JUNB*, giving rise to polysome size reduction and translation repression. **f** lncRNAs also act as miRNA sponges, leading to derepression of miRNA targets. As indicated, lncRNA *linc*-*MD1* “sponges” miR-133 and miR-135, antagonizing the miRNA-mediated translation suppression

In the nucleus, lncRNAs are implicated in posttranscriptional regulatory steps, including pre-mRNA splicing, mRNA capping, polyadenylation, and nuclear export. Pre-mRNA splicing is a key process to increase proteome diversity in higher eukaryotes [18]. The lncRNA metastasis-associated lung adenocarcinoma transcript 1 (*MALAT1*) has been proposed to regulate alternative splicing (AS) by modulating the distribution of active serine/arginine-rich (SR) proteins in nuclear speckle domains. SR proteins are essential splicing factors that function in both constitutive splicing and AS [19, 20]. Previous studies indicated that *MALAT1* modulated AS of endogenous pre-mRNAs by regulating SR splicing factors phosphorylation, as well as altering the distribution and ratio of phosphorylated versus non-phosphorylated pools of SR proteins (Fig. 2a). These changes may lead to alterations in the expression of specific isoforms of proteins in cells [21].

Another example of involvement of lncRNA in AS is *sno*-*lncRNA*, a class of nuclear-enriched intron-derived lncRNAs transcribed from a critical region of chromosome 15 (15q11-q13). This region is specifically deleted in Prader–Willi Syndrome (PWS). *sno*-*lncRNAs* are flanked by snoRNA sequences at both ends [22]. Studies have demonstrated that at least some of these *sno*-*lncRNAs* acted as molecular sinks of the splicing regulator Fox2, a member of the Fox family. *sno*-*lncRNAs* binded to Fox2 and altered the splicing patterns. Importantly, *sno*-*lncRNA* knockdown leaded to changes in Fox2-regulated splicing, while the overall gene expression levels were unaltered [23].

### lncRNAs in mRNA protection and decay

As an intermediate, mRNA carries information from genes to ribosomes for protein synthesis. However, it is unstable and the concentration of mRNA depends on the balance between the rates of synthesis and degradation [24]. lncRNAs are increasingly recognized as important modulators of both mRNA synthesis and degradation.

Functional characterization of the lncRNA *BACE1*-*AS* has revealed the action of lncRNAs in maintaining mRNA stability. *BACE1*-*AS* is a conserved antisense transcript partner of β-site amyloid precursor protein cleaving enzyme 1 (*BACE1*), which is a crucial enzyme in the pathogenesis of Alzheimer’s disease. When exposured to amyloid-β 1–42 (Aβ 1–42), which can induce oxidative stress, elevated *BACE1*-*AS* levels increase *BACE1* mRNA stability and generate additional Aβ 1–42 through a posttranscriptional feed-forward mechanism [25] (Fig. 2b).

Another type of lncRNA exerts its function by facilitating mRNA decay. Staufen 1 (STAU1) is a double-stranded RNA binding protein that binds to a subset of mRNAs and targets them for STAU1-mediated mRNA decay (SMD) [26]. Half-STAU1-binding site RNA (*1/2*-*sbsRNA*) is a polyadenylated lncRNA that induces mRNA decay by recruiting STAU1 to target mRNAs. STAU1-binding sites can be formed by imperfect base-pairing between an Alu element of an mRNA target of SMD and another Alu element in *1/2*-*sbsRNA*s. The *1/2*-*sbsRNA*-regulated mRNAs such as CUB-domain-containing protein 1 (*CDCP1*) mRNA and methylthioadenosine phosphorylase (*MTAP*) mRNAs can thus be degraded [27] (Fig. 2c).

### lncRNAs in translation activation and repression

mRNA translation is the final step of protein synthesis. Recent studies have demonstrated that some lncRNAs control protein synthesis by either post-transcriptional activation or repression of mRNA translation in the cytoplasm.

The nuclear-enriched lncRNA, *antisense* *Uchl1*, forms sense-antisense pairs by pairing with the ubiquitin carboxy-terminal hydrolase L1 (*Uchl1*) gene. Under stress conditions, *antisense* *Uchl1* lncRNA shuttles from the nucleus to the cytoplasm. It then binds the 5′end of the *Uchl1* mRNA and promotes the association of this overlapping sense protein-coding mRNA with active polysomes for translation [28] (Fig. 2d).

On the contrary, *lincRNA*-*p21* inhibits the translation of target mRNAs encoding β-catenin (*CTNNB1*) and JunB (*JUNB*) after HuR (also known as embryonic lethal abnormal vision 1, ELAVL1) is silenced. HuR is a ubiquitous RNA-binding protein that functions in cell proliferation, survival, and carcinogenesis, as well as in stress and immune responses [29, 30]. HuR exerts its functions mainly by interacting with a subset of mRNAs, and further increasing their stability and modulating their translation [31]. HuR also enhances the decay of *lincRNA*-*p21*. Therefore, in the absence of HuR, stable *lincRNA*-*p21* inhibits the translation of *CTNNB1* and *JUNB* mRNAs by enhancing their interaction with the translational repressor Rck, which may result in polysome size reduction and even ribosome “drop-off” [32] (Fig. 2e).

### lncRNAs in miRNA biology

microRNAs (miRNAs) are endogenous 19–23-nucleotide RNAs that negatively regulate gene expression at the post-transcriptional level. They interact with partially complementary sequences in the 3′-UTR of a target mRNA, leading to translational repression, mRNA cleavage, and mRNA decay [33, 34]. Recent reports have demonstrated that lncRNAs may prevent the repressive effects of miRNAs on their targets [35–37]. lncRNAs function as competing endogenous RNAs (ceRNAs) to sequester miRNAs, thereby protecting the target mRNAs from degradation [38].

*linc*-*MD1* has been implicated as a ceRNA that competes for shared miRNAs with mRNAs. Therefore, it can be regarded as an activator in mRNA translation. *Linc*-*MD1* “sponges” miR-133 and miR-135 to regulate the mRNA translation of mastermind-like-1 (*MAML1*) and myocyte-specific enhancer factor 2C (*MEF2C*), respectively (Fig. 2f). With the finding that both *MAML1* and *MEF2C* are critical genes for normal myogenic differentiation [39], *linc*-*MD1* is postulated to be involved in the control of myoblast differentiation [40]. The well-known lncRNA *H19* has been identified as a novel activator of the Wnt/β-catenin pathway by serving as a miRNA sponge. *H19* antagonizes the functions of miR-141 and miR-22, both of which are negative modulators of the Wnt/β-catenin pathway and osteogenesis. The presence of *H19* leads to the derepression of their shared target gene, β-catenin, and eventually promotes osteoblast differentiation [41].

## Modulation of osteoblastogenesis and osteoclastogenesis by lncRNAs

Mesenchymal stem cells (MSCs) have the potential to differentiate into multiple cell types, including osteoblasts, chondrocytes, adipocytes, and neurocytes [42]. Osteoclasts are derived from hematopoietic precursor cells of the monocyte-macrophage lineage. They are large, multinucleated, terminally differentiated cells, functioning as the sole bone-resorbing cells [43]. Skeletal development and adult bone remodeling depend on the coordinated function of osteoblasts and osteoclasts, which differentiate from precursor cells in the mesenchymal osteoblastic lineage [44] and the hematopoietic osteoclastic lineage [45], respectively.

The involvement of lncRNAs in the differentiation of MSCs into osteoblasts has been unveiled over the past decade (Table 1). Analysis of lncRNA expression profiles has revealed significant differences between untreated and bone morphogenetic protein 2 (BMP-2)-treated C3H10T1/2 MSCs [46]. In the study, the authors used BMP-2 to induce early osteoblastogenesis, and compared the differential expression profiles of lncRNA by microarray and bioinformatic approaches. Over 100 differentially expressed lncRNAs were identified. A subset of 24 lncRNAs was determined to concurrently change with their nearby coding genes, which are involved in osteoblastogenesis. For example, *mouselincRNA0231* and its nearby gene epidermal growth factor receptor (*EGFR*), which suppressed osteoblast differentiation via regulating Runx2 and Osterix, were downregulated after BMP-2 treatment. A similar correlation was observed between *NR_027652* and *mouselincRNA0243* with their respective nearby coding genes *DLK1* and *IL*-*5*, respectively. Another study demonstrated that anti-differentiation ncRNA (*ANCR*) regulated Runx2 expression and osteoblastogenesis. *ANCR* interacted with the enhancer of zeste homolog 2 (EZH2). The recruitment of *ANCR* with EZH2 catalyzed H3K27me3 in Runx2 gene promoter, resulting in the inhibition of Runx2 expression and subsequent osteoblastogenesis [47].

**Table 1:** Major lncRNAs associated with osteoblastogenesis and osteoclastogenesis, as well as skeletal and dental diseases

lncRNAs	Targets	Effects	References
*MouselincRNA0231*	Runx2, Osterix	Suppresses osteoblastogenesis	[46]
*ANCR*	Runx2	Inhibits osteoblastogenesis	[47]
*HIF1α*-*AS1*	HOXD10	Promotes osteoblastogenesis	[49]
*DANCR*	p-GSK-3β, β-catenin	Blocks odontoblast-like differentiation of hDPCs	[50]
*DANCR*	IL-6, TNF-α	Positively regulates osteoclastogenesis	[55]
*MEG3*	SLC39A1	Inhibits osteogenic differentiation of BMSCs	[65]
*Hotair*	PRC2, LSD1 complex	Repressor of skeletal malformation	[67]
*SOX9nc2*	SOX9	Promotes chondrogenesis	[71]
*H19*	COL2A1	Stimulates chondrocyte anabolism	[72, 73]
*lncRNA*-*POIR*	FoxO1	Positive regulator of osteogenic differentiation in periodontitis	[76]
*ANRIL*	*ADIPOR1*, *VAMP3*, *C110RF10*	Regulates risk variants of aggressive periodontitis	[77, 78]
*TUSC7*		Inhibits proliferation in osteosarcoma cells	[81, 82]
*MALAT*-*1*	SFPQ, PTBP2	Promotes proliferation, migration, or invasion in osteosarcoma cells	[83–85]
*LINC340*		Potentially involved in ameloblastoma	[89]

The function of the lncRNA hypoxia-inducible factor 1α-anti-sense 1 (*HIF1α*-*AS1*) in osteoblastogenesis was recently identified. *HIF1α*-*AS1* expression was significantly repressed after overexpression of the histone deacetylase sirtuin 1 (SIRT1), an important regulator of osteoblast differentiation [48]. Lower levels of SIRT1 gave rise to the upregulation of *HIF1α*-*AS1* in human bone marrow stem cells (BMSCs). Moreover, *HIF1α*-*AS1* knockout inhibited the expression of HOXD10 by interfering with acetylation, suggesting the potential role of *HIF1α*-*AS1* in the activation of osteoblastogenesis [49].

Attention has also been paid to the effects of lncRNAs on dentinogenesis (Table 1). Dentinogenesis shares many similarities with osteogenesis, and consists of multiple steps including odontoblast differentiation. Chen et al. showed that lncRNAs were involved in the odontoblast-like differentiation of human dental pulp cells (hDPCs) [50]. In their study, the expression of the differentiation-antagonizing lncRNA *DANCR* was considerably downregulated in a time-dependent manner in the process of hDPCs differentiation into odontoblast-like cells. Furthermore, mineralized nodule formation as well as the expression of dentin sialophosphoprotein and dentin matrix protein-1 was blocked after overexpression of *DANCR* in hDPCs. Upregulation of *DANCR* also decreased the expression levels of p-GSK-3β and β-catenin. These results reveal a role of *DANCR* in regulating the Wnt/β-catenin pathway and modulating dentin formation.

lncRNAs also play a regulatory role in osteoclastogenesis (Table 1). In one study, microarray analysis was performed to examine the expression profiles of lncRNAs at different stages of osteoclastogenesis. Then gene ontology analysis, pathway analysis, and lncRNA-mRNA co-expression network characterization showed the co-expression of multiple lncRNAs with tumor necrosis factor ligand superfamily member (*TNFSF*)12 and *TNFSF13* [51], factors involved in the differentiation of monocyte/macrophage precursor cells into osteoclasts [52, 53]. Circulating monocytes are directly involved in osteoclastogenesis by acting as osteoclast precursors [54]. The role of lncRNA *DANCR* in blood mononuclear cells has been studied. Overexpression of *DANCR* increased the secretion of IL-6 and TNF-α in blood mononuclear cells [55], both of which were inflammatory cytokines and important mediators of accelerated bone loss in osteoporosis [56, 57]. This suggests that *DANCR* can be a potential biomarker and regulatory element in circulating monocytes for osteoclastogenesis. However, further studies are needed to determine the underlying mechanisms of lncRNAs in osteoclastogenesis.

## lncRNAs in skeletal and dental diseases

lncRNAs not only play critical roles in various aspects of cellular biology but are also implicated in disease pathogenesis and progression. Several lncRNAs have been functionally associated with important pathogenic processes of cardiovascular diseases [58], diabetes [59], neurodegenerative disorders [60], immune response [61], as well as several types of cancer [62, 63]. However, the identity of lncRNAs in skeletal and dental diseases is not well known. Here, we summarize new findings in the functions of lncRNAs in these diseases (Table 1).

### Osteoporosis

Emerging evidence demonstrates the correlation of lncRNAs with osteoporosis, which is a common metabolic bone disorder [55]. Osteoporosis is characterized by reduced bone mineral density and increased incidence of fractures, resulting mainly from enhanced osteoclastic bone resorption activity outpacing bone formation by osteoblasts [64]. The sequence encoding lncRNA *DANCR* resides on human chromosome 4, located 54.8 kb upstream of *USP46* and 28.7 kb downstream from *ERVMER34*-*1* and the *ANCR* locus. As mentioned above, *DANCR* was upregulated in circulating monocytes of postmenopausal women with low bone mineral density, and could induce the expression of IL-6 and TNF-α [55]. These results suggest the important role of *DANCR* in the pathogenesis of osteoporosis and possibly as a biomarker for postmenopausal osteoporosis (PMOP). Another case is the involvement of lncRNA *MEG3* in the pathogenesis of PMOP [65]. In this study, *MEG3* expression was increased in BMSCs derived from PMOP patients and ovariectomized mice. *MEG3* directly bound to and activated miR-133a-3p, thereby inhibiting the expression of SLC39A1 (a direct target of miR-133a-3p), which was regarded as a positive regulator of osteogenic differentiation. Overexpression of *MEG3* inhibited osteogenic differentiation of BMSCs, which was markedly reversed by miR-133a-3p knockdown. These data indicate that lncRNAs participate in the pathogenesis of osteoporosis, which provide novel targets for the prevention and treatment of osteoporosis.

### Skeletal transformation

After its first identification in primary human fibroblasts [66], the lncRNA *Hotair* has also been found to be important in the embryonic patterning of the skeletal system [67]. Targeted deletion of *Hotair* resulted in lumbosacral homeotic transformation (6th lumbar vertebrae transform to 1th sacral vertebrae, L6 → S1) in a C57BL/6 mouse model. Moreover, malformation of the metacarpals and 4th caudal vertebrae was also observed. *Hotair* knockdown caused derepression of multiple *HoxD* cluster genes in embryos and tail tip fibroblasts. Insights into the molecular basis for the observed phenotypes revealed that *Hotair* acted *in trans* to bind both PRC2 and LSD1 complex. *Hotair* recruited them to hundreds of genomic sites to promote coordinated H3K27 methylation and H3K4 demethylation for gene silencing. However, in another study, the skeletal malformation indicated above was not detected after *Hotair* knockdown in a mixed CBAxBL/6 mouse model [68]. Whether the discrepancy in the phenotypic effects of *Hotair* knockdown is attributed to the different genetic background of animals needs further study.

### Osteoarthritis

Osteoarthritis (OA) is the clinical and pathological outcome of a range of disorders that results in structural and functional failure of synovial joints. While many risk factors (e.g., IL-1, IL-6, TNF-α, PGE2, MMPs) contribute to the onset of OA [69], the mechanism responsible for OA has not been fully elucidated. Recent studies have investigated the effects of lncRNAs on OA. Xing et al. reported that over 100 lncRNAs were up- or down-regulated in OA cartilage compared with normal cartilage based on microarray analysis. The increased expression of six lncRNAs (*HOTAIR*, *GAS5*, *PMS2L2*, *RP11*-*445H22.4*, *H19* and *CTD*-*2574D22.4*) in the microarray data was validated by real-time PCR [70], suggesting the regulatory potential of lncRNAs in OA. *SOX9nc2* is a cartilage-specific lncRNA which lies upstream of *SOX9* in the genome. Depletion of the *SOX9nc2* transcript by RNA interference prevented chondrogenesis and the expression of the transcription factor SOX9 [71]. Moreover, a significant correlation has been observed among the expression of lncRNA *H19*, miR-675, and *COL2A1* in OA cartilage. Co-upregulation of *H19*, *COL2A1*, and miRNA-675 was observed in chondrocytes under hypoxic conditions, which were known to stimulate chondrocyte anabolism. When chondrocytes were treated with inflammatory factors IL-1β and TNF-α to induce chondrocyte catabolism, the expression of *H19*, *COL2A1*, and miRNA-675 was significantly decreased [72]. In addition, Dudek et al. showed that inhibition of *H19* downregulated COL2A1, while overexpression of miR-675 rescued COL2A1 expression in *H19*-depleted human articular chondrocytes [73]. More work is needed to investigate the function and mechanisms of lncRNAs as key regulators of OA.

### Periodontitis

Evidence for the relationship between periodontitis and lncRNAs is emerging. Periodontitis is a common chronic inflammatory disease initiated by a group of bacterial pathogens in dental plaque. The inflammation extends deep into tissues, damages the connective tissue and alveolar bone around teeth, and eventually leads to tooth loss [74]. Microarray analysis of lncRNA expression profile revealed a total of 8925 differently expressed lncRNAs in chronic periodontitis tissues compared with adjacent normal tissues. Further subgroup analysis showed there were 589 enhancer-like lncRNAs, 238 HOX cluster lncRNAs, as well as 1218 lincRNAs. Based on the information, the function and mechanisms of lncRNAs associated with periodontitis needs further investigation [75]. The role of a crucial lncRNA related to periodontitis, *lncRNA*-*POIR*, has recently been investigated. *lncRNA*-*POIR* expression was significantly lower in periodontal mesenchymal stem cells (PDLSCs) from periodontitis patients (pPDLSCs) than that in human periodontal MSCs (hPDLSCs). Overexpression of *lncRNA*-*POIR* promoted osteogenic differentiation of pPDLSCs. Further study revealed that *lncRNA*-*POIR* acted as a ceRNA for miR-182, thus positively regulating expression of FoxO1. The inflammatory environment, which usually occurred in periodontitis, increased miR-182 expression through NF-κB pathway, finally resulted in an imbalance in the *lncRNA*-*POIR*-miR-182 regulatory network [76]. The association between periodontitis and another lncRNA *ANRIL* has also been reported. *ANRIL* is the first shared genetic risk factor of coronary artery disease and aggressive periodontitis [77]. Bochenek et al. demonstrated that *ANRIL* knockdown resulted in repression of three genes *ADIPOR1*, *VAMP3*, and *C11ORF10*. Exploration of the identified genes highlighted a region upstream of *VAMP3* within *CAMTA1* (rs10864294) to be associated with increased risk of coronary artery disease and aggressive periodontitis [78]. These studies indicate the potential of lncRNAs as diagnostic biomarkers and targets for the treatment of periodontitis.

### Osteosarcoma

Differences in the expression of lncRNAs in different types of tumors have been well documented [79, 80]. This finding promoted interest in addressing the potential of lncRNAs in skeletal tumors. Osteosarcoma is the most common primary malignant tumor of bone with cytogenetic complexity. The lncRNA *TUSC7* (tumor suppressor candidate 7), previously named as *LOC285194*, was significantly downregulated in osteosarcomas. The decreased expression of *TUSC7* was due to copy number loss of the genomic region on chr3q13.31. Depletion of *TUSC7* promoted proliferation and inhibited apoptosis in osteosarcoma cells. *TUSC7* suppression also increased osteosarcoma growth in a mouse model, and was correlated with poor survival of osteosarcoma patients [81, 82]. In addition, recent studies revealed that the lncRNA *MALAT1* was dysregulated in multiple malignant tumors, including osteosarcoma. Knockdown of *MALAT1* decreased proliferation, migration, and induced apoptosis in osteosarcoma. *MALAT1* knockdown significantly inhibited PI3K/AKT and RhoA/ROCK signaling pathway. High expression of *MALAT1* was closely correlated with pulmonary metastasis in patients with osteosarcoma [83, 84]. Interestingly, Fang et al. demonstrated that downregulation of *MALAT1* induced by high dose of 17β-Estradiol promoted the binding of SFPQ to oncogene PTBP2, therefore affecting proliferation, migration or invasion in osteosarcoma cells [85]. Further discussion of lncRNAs in osteosarcoma can be found elsewhere [86, 87].

### Ameloblastoma

Ameloblastoma is a benign but locally invasive odontogenic tumor of the jaws [88]. It often results in facial deformity and significant morbidity because of its high rate of recurrence and requirement for radical surgery. Considerable efforts have been made to clarify the underlying molecular mechanisms and actions of lncRNAs in ameloblastoma. The ncRNA expression profile of ameloblastoma was characterized in a well-defined ameloblastoma cohort. In this study, whole transcriptome profiling by microarray followed by real-time PCR assays validated five highly associated ncRNAs, including the lncRNA *LINC340* (also known as *CASC15*). However, whether LINC340 is a prognostic and therapeutic marker that can improve the treatment of ameloblastoma requires further investigation [89].

## Conclusions

Over the past decade, extensive research has established that lncRNAs play important roles in diverse cellular processes. Moreover, the molecular mechanisms by which lncRNAs exert their functions have been largely elucidated. These discoveries have promoted investigators in the skeletal and dental fields to address the potential role of lncRNAs in regulating the differentiation and function of bone cells as well as in the pathogenesis of skeletal and dental diseases. However, whereas a few studies have revealed the functional role of some lncRNAs, most of the results have merely demonstrated an association of lncRNAs with either bone cell biology or the development of some skeletal and dental diseases. Hence, future investigations should focus on further establishing the functional links between lncRNAs and hard tissue diseases and elucidating the underlying molecular mechanisms. A better understanding of the regulatory roles and molecular mechanisms of lncRNAs in skeletal and dental diseases may identify new biomarkers for diagnosis and novel therapeutic targets for these disorders.

## Abbreviations

ANCR: anti-differentiation noncoding RNA
AS: alternative splicing
Aβ 1-42: amyloid-β 1-42
BACE1: β-secretase-1
BMSC: bone marrow stem cell
BMP-2: bone morphogenetic protein 2
CDCP1: CUB-domain-containing protein 1
ceRNA: competing endogenous RNA
DANCR: differentiation-antagonizing long noncoding RNA
EGFR: epidermal growth factor receptor
EZH2: enhancer of zeste homolog 2
hDPC: human dental pulp cell
HIF1α-AS1: hypoxia-inducible factor 1α-anti-sense 1
HOX: homeobox
hPDLSC: human periodontal mesenchymal stem cell
H3K4me3: trimethylation of lysine 4 on histone H3
H3K9me3: trimethylation of lysine 9 on histone H3
H3K27me3: trimethylation of lysine 27 on histone H3
lincRNA: long intergenic noncoding RNA
lncRNA: long noncoding RNA
LSD1: lysine-specific demethylase 1
MALAT1: metastasis-associated lung adenocarcinoma transcript 1
MAML1: mastermind-like-1
MEF2C: myocyte-specific enhancer factor 2C
miRNA: microRNA
MSC: mesenchymal stem cell
MTAP: methylthioadenosine phosphorylase
ncRNA: non-protein-coding RNA
OA: osteoarthritis
PDLSC: periodontal mesenchymal stem cell
PMOP: postmenopausal osteoporosis
Pol II: polymerase II
pPDLSC: patient periodontal mesenchymal stem cell
PRC2: polycomb repressive complex 2
PWS: Prader–Willi Syndrome
rRNA: ribosomal RNA
RT-PCR: real-time polymerase chain reaction
SIRT1: sirtuin 1
SMD: STAU1-mediated mRNA decay
snoRNA: small nucleolar RNA
snRNA: small nuclear RNA
SR protein: serine/arginine-rich protein
STAU1: Staufen 1
TNFSF: necrosis factor ligand superfamily
tRNA: transfer RNA
Uchl1: ubiquitin carboxy-terminal hydrolase L1
XIST: X inactive-specific transcript
1/2-sbsRNA: half-STAU-1-binding site RNA
3′-UTR: 3′-untranslated region
